# Dynamic effects of lower limb mechanical-axis shift on medial meniscus posterior root radial tear

**DOI:** 10.3389/fbioe.2026.1925268

**Published:** 2026-10-14

**Authors:** Chengyue Yu, Shuming Cao, Kexin Liu, Jingyu Zhang, Wenjun Zhao, Xiaoyuan Duan, Bo Liu, Lupeng Wang, Zhao Liu, Yongxin Huo, Tao Zhang, Guosheng Xing, Weiguo Xu

**Affiliations:** 1 Tianjin Hospital, Tianjin University, Tianjin, China; 2 Weifang People’s Hospital, Weifang, Shandong, China; 3 Changchun Institute of Optics, Fine Mechanics and Physics, Chinese Academy of Sciences, Jilin, China; 4 The Second Hospital of Tangshan, Tangshan, Hebei, China

**Keywords:** finite element dynamics, mechanical-axis shift, MMPRRT, osteoarthritis, peak contact pressure

## Abstract

**Introduction:**

Lower limb mechanical alignment and medial meniscus posterior root radial tears (MMPRT) are considered risk factors for increased knee joint compartment loading, which may promote the development of osteoarthritis. MMPRT accounts for 21.5% of medial meniscus tears, with the medial meniscus posterior root radial tear (MMPRRT) being the most common type. Its occurrence is closely associated with dynamic loading of the knee joint during movement. This study aimed to establish a novel dynamic model of mechanical-axis shift and to investigate the biomechanical effects of mechanical-axis shift and tear length on MMPRRT under dynamic loading conditions.

**Methods:**

One healthy male volunteer was recruited. The volunteer had a neutral mechanical alignment confirmed by full-weight-bearing lower limb radiographs (R_HKA 0.44°, R_mMPTA 88.61°, R_mLDTA 87.07°, R_mLDFA 88.77°, R_JLCA 0.30°). CT and MRI data of the right knee were acquired. A finite element model of the knee joint at neutral alignment (0°) was established. A novel method for constructing mechanical-axis shift was proposed: only the load application point at the femoral end was translated in the medial–lateral direction to simulate mechanical-axis shifts, without directly modeling anatomical malalignment or altering the basic geometry and ligament tension of the knee joint. A total of six finite element models of MMPRRT under different mechanical-axis shift conditions were established, including medial mechanical-axis shifts equivalent to 3°, 6°, and 9°, and lateral mechanical-axis shifts equivalent to 3° and 6°. Dynamic analyses were performed using ISO-14243-3 gait loading. The finite element model at neutral alignment (0°) was validated using one cadaveric knee specimen under one static loading condition (1150 N), with the medial compartment contact area as the validation metric.

**Results:**

Under static loading, the error in medial compartment contact area between the finite element model and the cadaveric experiment was 5.72% for the intact meniscus condition. The peak contact pressure of each knee structure exhibited two peaks at 14% and 44% of the gait cycle. At neutral alignment (0°) and 14% of the gait cycle, the tear gap of the unstable posterior root radial tear was 48.75% larger than that of the stable tear. The maximum meniscal extrusion displacement was 2.69 mm, occurring at 44% of the gait cycle. A medial mechanical-axis shift elevated the medial peak contact pressure and prolonged the duration of high overall meniscal displacement, whereas a lateral mechanical-axis shift reduced the medial peak contact pressure and displacement.

**Discussion:**

This study established a dynamic finite element model of the knee joint with mechanical-axis shift and investigated the effects of mechanical-axis shift and tear length on MMPRRT biomechanics. The results showed that a medial mechanical-axis shift was associated with increased medial compartment loading, meniscal displacement, and tear gap, whereas a lateral mechanical-axis shift was associated with reduced medial loading. These biomechanical associations suggest that mechanical-axis shift may influence the local mechanical environment of MMPRRT. However, the clinical relevance of these findings requires further validation through prospective clinical studies incorporating multi-subject models and patient data.

## 1 Introduction

The meniscus plays a crucial role in maintaining normal knee function by stabilizing the tibiofemoral joint, providing lubrication, and absorbing shock (Fox et al., 2012; Berk et al., 2024). Meniscal tears are a common knee injury (Mao et al., 2025). Owing to the presence of hoop collagen fibers, the meniscus converts axial loads into hoop stresses (Jiang et al., 2020). When a meniscal tear occurs, particularly a radial tear, the continuity of these hoop fibers is disrupted (Kedgley et al., 2019). This disruption leads to increased local pressure on the meniscus and articular cartilage, accelerates wear, and may further contribute to the development of osteoarthritis (Zhang et al., 2019). Medial meniscus posterior root tears (MMPRT) are a common subtype of meniscal injury, accounting for approximately 21.5% of all medial meniscus tears (Kamatsuki et al., 2023). Among these, the radial tear pattern at the medial meniscus posterior root (MMPRRT) is the most frequently encountered subtype.

Therefore, research on this type of injury should not be limited to static analysis; dynamic analysis is essential. Unlike static analysis, which only reflects the instantaneous mechanical state at a specific flexion angle, dynamic analysis can simulate real-time changes in knee joint contact forces and joint kinematic parameters throughout the entire gait cycle (Xu et al., 2022; Yang et al., 2023; Mao et al., 2025). Static models are unable to capture several key pieces of information: (1) the time-dependent changes in contact pressure and the bimodal distribution pattern during the gait cycle; (2) the cyclic opening and closing behavior of the tear gap under dynamic loading and its potential impact on tear propagation; (3) the phase-dependent dynamic changes in meniscal extrusion displacement, which are directly related to the failure mode of the hoop mechanism. These dynamic data may provide mechanical references for future clinical investigations, including the identification of high-risk movement phases during postoperative rehabilitation, the assessment of tear stability and healing potential for different tear sizes, and the evaluation of surgical timing and postoperative outcomes. However, these potential clinical applications require further validation. In recent years, dynamic finite element analysis has been increasingly applied to evaluate the effects of dynamic loading on meniscal tears, with results indicating that dynamic loading conditions can reveal a more realistic joint mechanical environment, which is of great importance for understanding the mechanical mechanisms underlying joint degeneration associated with MMPRRT (Wang et al., 2022; Yang et al., 2023).

Knee malalignment increases contact pressure in the corresponding compartment (Berk et al., 2024). This creates an unfavorable biomechanical environment. It has been established as an independent risk factor for medial compartment overload. It also contributes to the onset and progression of knee osteoarthritis (Willinger et al., 2019; Willinger et al., 2020). When MMPRRT coexists with malalignment, the hoop stress transmission function of the meniscus is further compromised. This may accelerate alignment deterioration and joint degeneration, forming a vicious cycle. Previous biomechanical studies have demonstrated that varus alignment increases peak contact pressure in the medial compartment. Valgus alignment helps reduce medial compartment loading (Mina et al., 2008; Willinger et al., 2019; Willinger et al., 2020). However, these findings are derived from static or quasi-static experiments. Systematic investigations into the dynamic mechanical responses of MMPRRT under malaligned knee conditions remain lacking.

The contributions of this study are summarized in three aspects. First, a loading-point shift method was proposed to simulate mechanical-axis shift. This method enables dynamic finite element analysis of medial and lateral mechanical-axis shifts within the same anatomical model. It also avoids confounding effects from inter-individual geometric variation. Second, the time-dependent changes in contact pressure, tear gap, and meniscal extrusion displacement of MMPRRT were revealed during the dynamic gait cycle. This addresses the limitation of static analysis, which cannot capture time-related mechanical characteristics. Third, the dose-response relationship between mechanical-axis shift magnitude and MMPRRT mechanical parameters was quantitatively assessed. This provides a preliminary mechanical basis for exploring alignment correction thresholds.

Based on the above methods, this study aimed to establish a dynamic knee model with mechanical-axis shift and to investigate the biomechanical effects of mechanical-axis shift and tear length on MMPRRT under dynamic loading. We hypothesized that MMPRRT increases meniscal contact pressure and medial compartment loading under dynamic loading. A medial mechanical-axis shift exacerbates the mechanical environment of the meniscal root tear, while a lateral mechanical-axis shift has a relatively minor effect.

## 2 Materials and methods

### 2.1 Neutral alignment knee finite element model

A 22-year-old male volunteer was recruited (height: 170 cm; BMI: 23). The volunteer had neutral mechanical alignment. Alignment parameters were measured on full-length standing radiographs: R-HKA 0.44°, R-mMPTA 88.61°, R-mLDTA 87.07°, R-mLDFA 88.77°, and R-JLCA 0.30°. The volunteer had no history of medical or surgical disease. There was no history of knee injury or surgery. Physical examination and radiography excluded knee joint pathology. Written informed consent was obtained from the volunteer. All methods were performed in accordance with the Declaration of Helsinki. The experimental protocol was approved by the Ethics Committee of Tianjin Hospital, Tianjin University (IRB: 2024 Medical Ethics Bureau Pre-020).

The detailed construction procedures can be found in Yu et al. (2025). We selected the right knee of this volunteer for magnetic resonance imaging (MRI) (SIGNA 3.0T; GE, Las Vegas, NV, United States). The slice thickness was 1 mm. A total of 250 MRI images were acquired to extract soft tissues, including the menisci, ligaments, and articular cartilage. Computed tomography (CT) of the same knee was performed using a Discovery CT 750 HD system (GE, United States). The slice interval was 0.625 mm. CT images were used to reconstruct bony structures, including the femur, tibia, fibula, and patella. The DICOM images from both CT and MRI were imported into MIMICS software (Materialise, Leuven, Belgium) for three-dimensional knee reconstruction. The reconstructed structures included the femur, tibia, fibula, patella, menisci, and cartilage (femoral, tibial, fibular, and patellar cartilage), as well as ligaments, including the anterior cruciate ligament (ACL), posterior cruciate ligament (PCL), medial collateral ligament (MCL), lateral collateral ligament (LCL), patellar tendon, and quadriceps tendon. All bony and soft tissue structures were manually segmented from the images by an experienced orthopedic clinician and a radiologist.

Each model of the knee was smoothed, refined, and surface-fitted using Geomagic Studio software (Geomagic Solutions, Cary, NC, United States). The processed models were then imported into SolidWorks software. Soft tissues generated from MRI images and bony structures from CT images were assembled to construct a three-dimensional knee model (Figure 1a). Previous studies have reported that contact pressure has low sensitivity to changes in cartilage thickness (Li et al., 2001; Beillas et al., 2007; Segal et al., 2009; Kazemi et al., 2013). Therefore, cartilage thickness parameters were set uniformly in this study.

**FIGURE 1:**
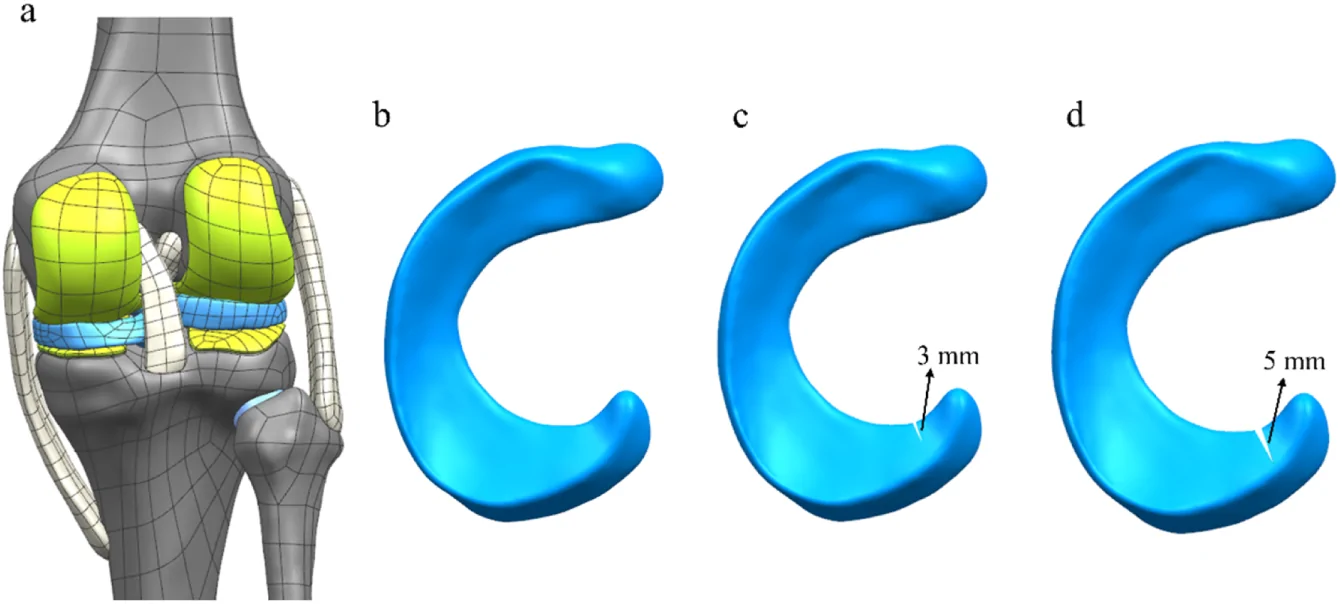
Knee joint model and meniscus tear model. **(a)** 3D knee joint model; **(b)** Intact medial meniscus; **(c)** Stable radial meniscus tear at the medial posterior root; **(d)** Unstable radial meniscus tear at the medial posterior root.

The three-dimensional knee geometric model was imported into Abaqus 6.20 (SIMULIA, France) for analysis using the explicit solver. A complete knee finite element model was established through meshing, material assignment, and boundary condition settings. Tetrahedral elements (C3D10) were used in this model. Although hexahedral elements provide higher accuracy, they are difficult to generate due to the irregular geometry of the knee joint. Mesh convergence tests showed that the error in peak tibial cartilage stress was within 2%. This accuracy was acceptable for establishing multiple mesh models with varying densities. Figure 2 illustrates the relationship between mesh size and convergence of tibial cartilage stress. A bone element size of 2.5 mm and an average soft tissue element size of 1 mm were adopted. This mesh configuration ensured result convergence while maintaining computational efficiency. The neutral knee model consisted of 773,394 tetrahedral elements and 201,499 nodes.

**FIGURE 2:**
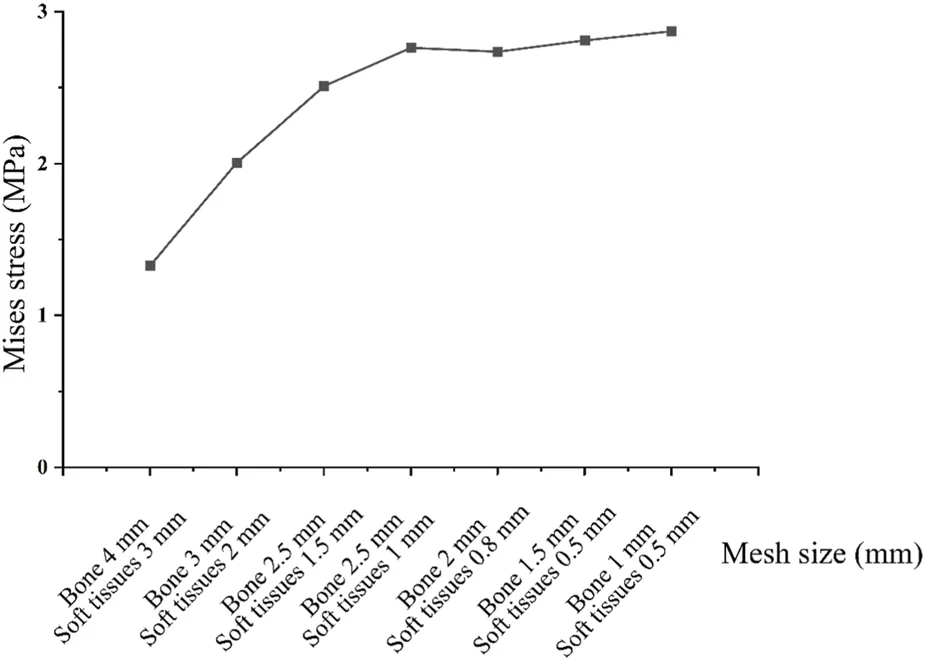
Mesh convergence study.

### 2.2 Material properties

Given that bone stiffness is substantially higher than that of soft tissues and that bone deformation has a negligible effect on joint contact mechanics, the bony structures were modeled as rigid bodies. Articular cartilage was modeled as an isotropic, hyperelastic, and nearly incompressible material using the Yeoh form (Robinson et al., 2016); the corresponding material parameters are listed in Table 1. The menisci were defined as transversely isotropic materials (Kedgley et al., 2019; Jiang et al., 2020; Steineman et al., 2020; Daszkiewicz and Łuczkiewicz, 2021; Wang et al., 2021; Wang et al., 2022), with a hoop modulus significantly higher than the axial modulus (see Table 2 for parameters). Ligaments were modeled as nearly incompressible, nonlinear, hyperelastic, and transversely isotropic materials based on a Neo-Hookean formulation (Luczkiewicz et al., 2016); their material parameters are provided in Table 3.

**TABLE 1:** Material parameters of cartilage (Robinson et al., 2016).

Parameter	Femoral cartilage	Tibial cartilage
$C_{10}\left(MPa\right)$	1.4	2.0
$C_{20}\left(MPa\right)$	3.6	4.5
$D_{1}\left(MPa^{-1}\right)$	0.0739	0.0517

**TABLE 2:** Meniscus material parameters (Luczkiewicz et al., 2015; Wang et al., 2021).

Component	$E_{\theta }\left(MPa\right)$	$E_{r},E_{z}\left(MPa\right)$	$\nu_{r\theta },\nu_{z\theta }$	$\nu_{rz}$	$G_{r\theta },G_{z\theta }\left(MPa\right)$	$G_{rz}\left(MPa\right)$
Lateral meniscal	120	20	0.3	0.2	57.7	8.33
Medial meniscal	120	20	0.3	0.2	57.7	8.33

*E* -Young modulus; *v* - Poisson ratio; *G* - Shear modulus (The subscripts θ , r , and z are hoop, radial, and axial constants directions, respectively).

**TABLE 3:** Material constants of the ligaments (Luczkiewicz et al., 2016).

Component	$C_{1}\left(MPa\right)$	$D_{1}\left(MPa^{-1}\right)$	$C_{3}\left(MPa\right)$	$C_{4}$	$C_{5}\left(MPa\right)$	$\lambda^{*}$
ACL	1.95	0.01366	0.0139	116.22	535.039	1.046
LCL	1.44	0.00252	0.57	48.0	467.1	1.062
MCL	1.44	0.00252	0.57	48.0	467.1	1.062
PCL	3.25	0.0082	0.1196	87.178	431.063	1.035

Constants $C_{1}$ and $D_{1}$ -Neo-Hookean constants, $C_{3}$ - the exponential stress, $C_{4}$ - the rate of collagen uncramping $C_{5}$ - the elastic modulus of the straightened collagen fibers, $\lambda^{*}$ - predetermined fiber stretch value beyond which collagen fibers straighten.

### 2.3 Model sensitivity analysis

The material properties of each component of the knee joint and the friction coefficient at the articular interfaces are among the representative uncertain factors in knee joint modeling. This study therefore performed a sensitivity analysis on the effects of meniscal material, ligament material, cartilage material, friction coefficient, and cartilage Poisson’s ratio (Table 4). The results showed that meniscal material, ligament material, and cartilage material had minor effects on the peak contact pressure of the joint. The knee joint model exhibited low sensitivity to these parameters (Figure 3). The sensitivity analysis revealed that the peak contact pressure increased with increasing Poisson’s ratio. The maximum variation in peak contact pressure due to changes in Poisson’s ratio was 25%. Regarding the friction coefficient, the peak contact pressure in Figure 3 initially decreased and then increased with varying friction coefficient. This fluctuation was speculated to be associated with the nonlinear characteristics of the complex contact mechanics of the knee joint. The friction coefficient affects not only the tangential forces at the contact interface but also the peak pressure indirectly by altering the stress distribution in the contact region. According to the sensitivity analysis results, the peak contact pressure varied from −7.80% to 24.76% when the friction coefficient changed from 0.02 to 0.1. These findings indicated that Poisson’s ratio and the friction coefficient showed a certain degree of sensitivity to the absolute value of peak contact pressure. However, all conditions in this study were compared based on the same model framework and identical material parameter settings. The influence of these parameters on the trend of the results was therefore limited. Overall, the present model demonstrated good robustness under various parameter variations.

**TABLE 4:** Sensitivity analysis.

Component	Parameters		
Meniscus	Hgo	Transversely isotropic materials	
Ligament	Neo-Hookean	Isotropic materials E=6 MPa , ν=0.4	
Cartilage	Yeoh	OA materials	Neo-Hookean
		Tibia OA: $C_{10}=1.1\;MPa,C_{20}=2.7\;MPa,D_{1}=0.094\;MPa^{-1}$ Femur OA: $C_{10}=1.2\;MPa,C_{20}=2.2\;MPa,D_{1}=0.0862\;MPa^{-1}$	$\begin{array}{l} C_{10}=0.86\;MPa,\\ D_{1}=0.048\;MPa^{-1} \end{array}$
f	0.02//0.035/0.05/0.08/0.1		
v	0.45/0.46/0.47/0.48/0.49		

**FIGURE 3:**
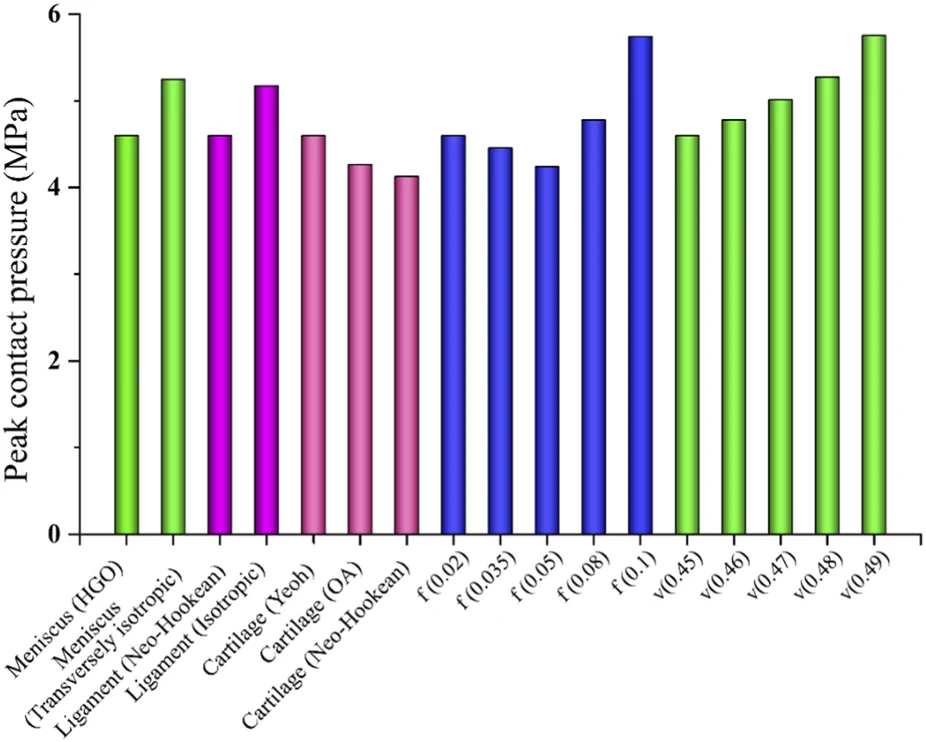
Peak contact pressure of the joint cavity under different material properties, friction coefficients, and cartilage Poisson’s ratios.

### 2.4 Model setup

In dynamic simulations, the mass properties of materials are essential for accurate calculation of inertial forces. The material densities of the knee joint structures used in this study are summarized in Table 5. All simulations were performed using ABAQUS software (SIMULIA, France) with its dynamic explicit solver. The total simulation time was set to 1.0 s. The global time increment was set to 1 × 10^−6^ s. To ensure the numerical accuracy of the explicit dynamic solutions, the following quality control measures were implemented for all dynamic simulations in this study. (1) Mass scaling. No mass scaling was used in the dynamic simulations conducted in this study. All structures were assigned real density values to preserve the physical authenticity of inertial forces. (2) Energy monitoring. The complete meniscus model at the neutral position (0°) was selected as the representative condition. The ratio of kinetic energy (KE) to internal energy (IE) was monitored throughout the full gait cycle. The results showed that this ratio remained below 5% during the entire gait cycle. This indicated that inertial artifacts did not dominate the computational results. (3) Convergence analysis of time increment step. Based on the global time increment of 1 × 10^−6^ s, a convergence verification of the time step was performed. The time step was refined to 1 × 10^−7^ s. The change in the key output variable (peak contact pressure) was less than 2%. This confirmed that the time increment of 1 × 10^−6^ s met the requirement for computational accuracy while maintaining a balance between computation time and precision. This setting also satisfied the CFL stability limit for the explicit solver.

**TABLE 5:** Densities of the knee joint structures.

Structure	Density (g/cm^3^)	References
Cortical bone	2.00	Asgari and Keyvanian (2019), Kiapour et al. (2014)
Cancellous bone	0.62	Bayraktar et al. (2004)
Articular cartilage	1.00	Kiapour et al. (2014), Makinejad et al. (2013), Beillas et al. (2004)
Ligaments	1.00	Dhaher et al. (2010), Penrose et al. (2002)
Meniscus	1.50	Kiapour et al. (2014), Asgari and Keyvanian (2019)

The boundary conditions were defined as follows. The distal fibula was fully fixed in the dynamic analysis. The tibia was allowed to move in the anterior-posterior direction and rotate in the internal-external direction. The femur was free in the axial direction. The femur was also free in varus-valgus rotation, internal-external rotation, and flexion angle. At the neutral position, the femoral loading point was set at the center of rotation of the femur. This point was located 5 mm medial to the midpoint of the most distal points of the two femoral condyles. The tibial loading point was set at the midpoint of the medial and lateral tibial plateaus. Frictional contact was defined between the components of the knee joint. A tangential sliding friction coefficient of 0.02 was assigned between cartilage-cartilage interfaces and between cartilage-meniscus interfaces (Daszkiewicz and Łuczkiewicz, 2021). Rigid connections were defined between cartilage and bone tissues and between ligaments and bone tissues.

The loading protocol was based on the ISO-14243-3 standard for artificial knee joints (Figure 4). This standard was originally developed for wear testing of knee prostheses. However, the loading curves (axial force, flexion angle, anterior-posterior displacement, and internal-external rotation angle) were derived from statistical averages of knee joint loading and kinematic data during the human gait cycle. The loading waveforms were highly consistent with the physiological loading patterns of natural knee joints during normal walking. This standard has been widely applied in dynamic finite element analyses of natural knee joints. The body weight of the subject in this study was similar to the reference population of this standard. Therefore, no individual body weight scaling was performed.

**FIGURE 4:**
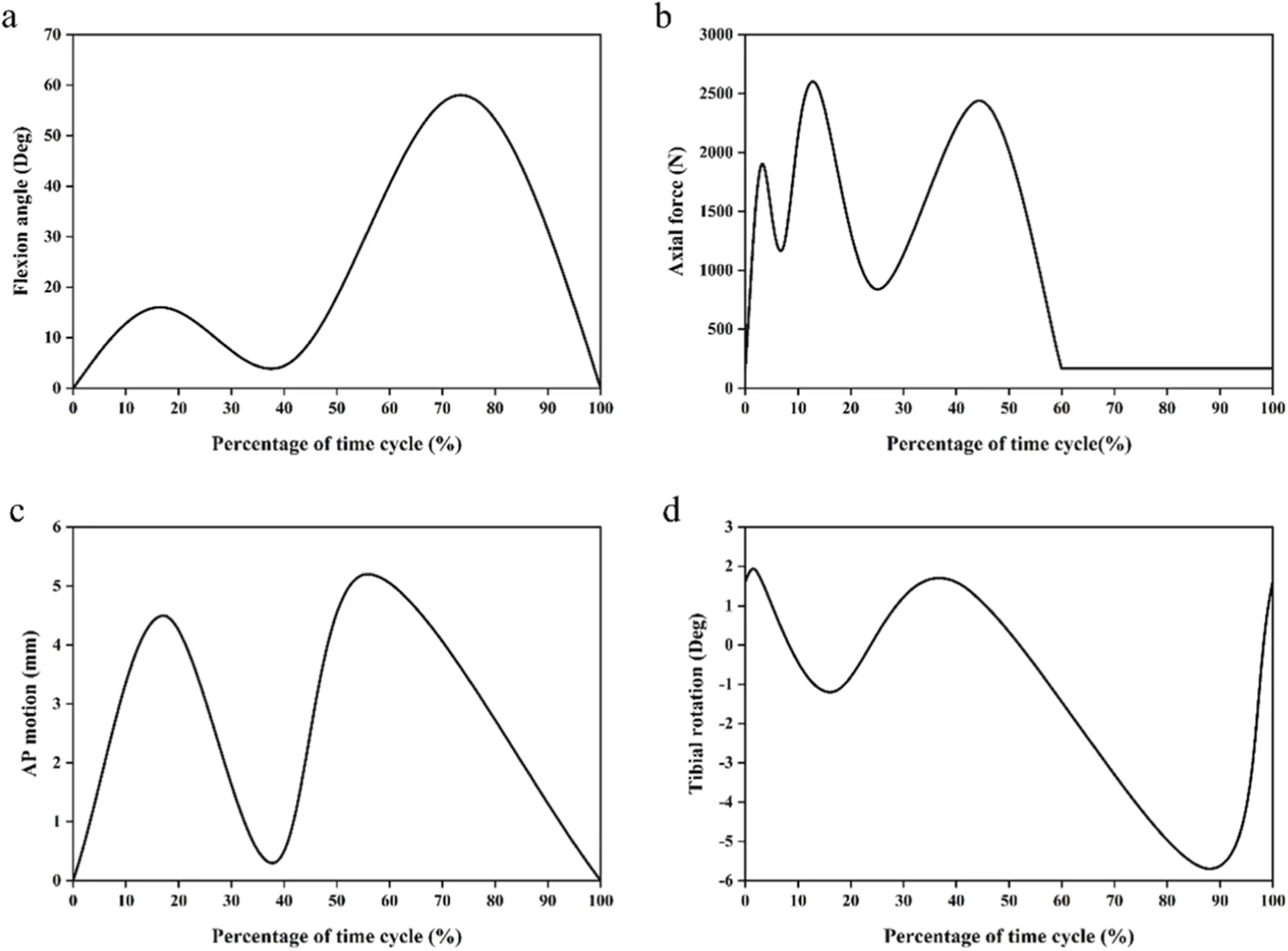
Input function of finite element model based on ISO 14243-3 gait cycle (Xu et al., 2022). **(a)** Flexion angle; **(b)** Axial force; **(c)** AP motion; **(d)** Tibial rotation.

The quadriceps tendon and patellar ligament were included in the model as three-dimensional solid structures. They were attached to the anatomical insertion regions on the femur and tibial tuberosity, respectively. In this model, no active muscle contraction forces were applied to these structures. They were included as passive constraint structures in an initial stress-free state to participate in joint kinematic restriction. Their primary function was to restrict excessive anterior translation and internal-external rotation during dynamic gait simulation through constraint forces generated by passive stretching. This enhanced the passive stabilization effect of the tibiofemoral joint.

### 2.5 Establishment of the MMPRRT model

In varus knees, the posterior root of the medial meniscus is more susceptible to tearing. Therefore, the medial meniscus was selected to construct the meniscal radial tear model. Kedgley et al. (Kedgley et al., 2019) defined a stable radial tear as a tear with a length less than one-third of the meniscal width. In Solidworks software, the posterior root of the intact medial meniscus was sectioned. The tear lengths were set to 3 mm and 5 mm, respectively. The width at the posterior root of the meniscus was approximately 10 mm. These tear lengths correspon4ded to stable and unstable radial tears, respectively.

The geometric definition of the tear was as follows. The tear originated at a point 7 mm from the center of the posterior root attachment of the medial meniscus. It extended radially toward the free edge of the meniscus. The tear was defined as a full-thickness lesion. The tear width was set to slightly greater than zero. This was achieved by geometrically creating two opposing faces with no finite gap between them. The contact property at the tear surfaces was defined as frictionless contact. Separation was allowed while penetration was prevented. Under this definition, the circumferential fibers were completely interrupted at the tear site. The fibers remained partially continuous on both sides of the tear. The tear geometry is shown in Figures 1c,d.

### 2.6 Development of mechanical-axis shift models

Dynamic loading was applied at six mechanical-axis shift conditions: neutral (0°), medial shift (3°, 6°, and 9°), and lateral shift (3°and 6°). The loading point for the medial and lateral shift conditions was shifted medially or laterally relative to the neutral loading point. The offset distance was derived as follows Figure 5.

**FIGURE 5:**
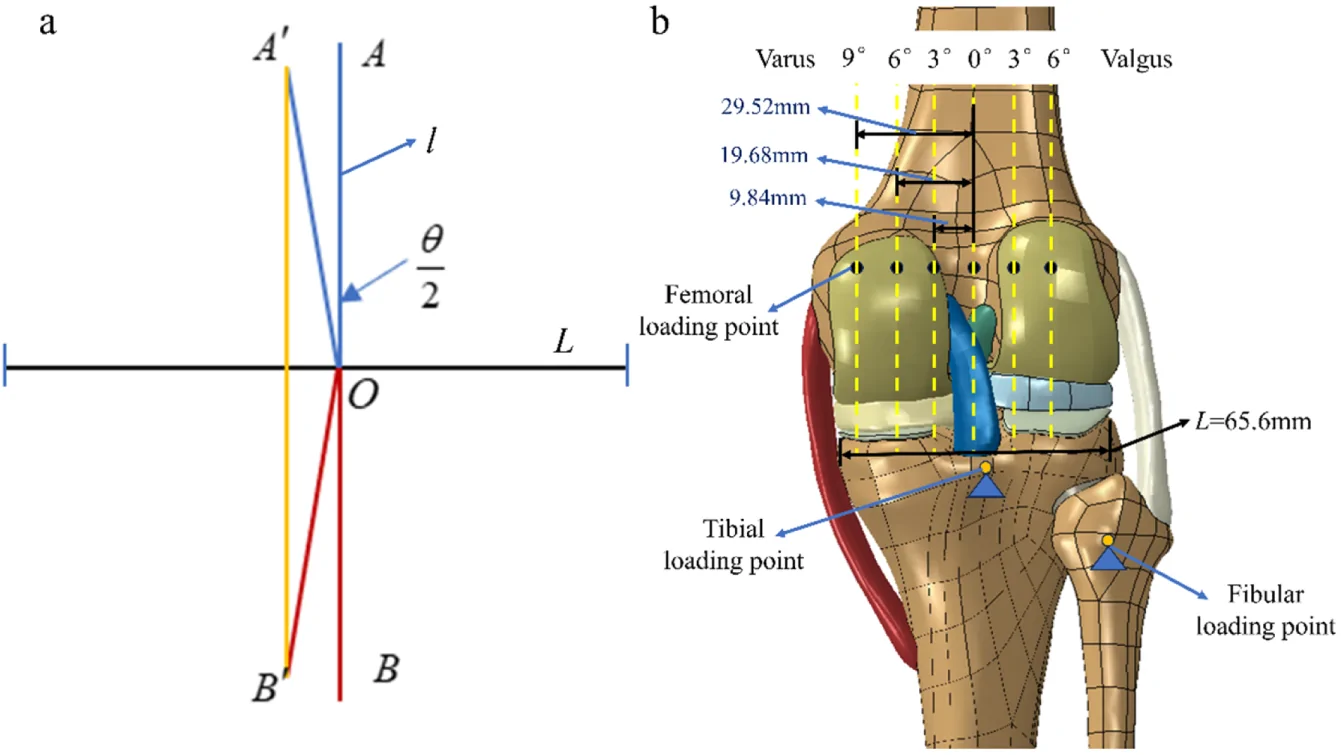
Schematic diagrams of equivalent varus angle and mechanical-axis shift, and loading point translation. **(a)** Schematic diagram of equivalent varus angle and mechanical-axis shift; **(b)** Schematic diagram of loading point translation.

As shown in Figure 5a, AB is the neutral lower limb force line, ∠AOB represents the knee joint HKA angle, when the equivalent varus angle is θ °, $A\to A^{\prime }$ , $B\to B^{\prime }$ . The relationship between the angle and the distance of translation is shown in Equation 1.

$$\cos\left(90-\frac{\theta }{2}\right)=\frac{d}{l}$$ (1)

Where: d represents the distance of translation, and l represents the femoral or tibia length. $\frac{d}{L}$ represents the ratio of the medial translation distance d to the tibial plateau width L . When θ is 1°, $\frac{d}{L}\approx 0.05$ . Here, for 1° of equivalent varus, the medial displacement of the force line is 0.05L .

The loading point at the neutral position (0°) was shifted medially to represent 3°, 6°, and 9° of equivalent varus, respectively. The loading point at the neutral position (0°) was shifted laterally to represent 3° and 6° of equivalent valgus, respectively. The loading positions are shown in Figure 5b. In the above derivation, *L* was defined as the maximum distance between the medial and lateral edges of the tibial plateau, which represented the tibial plateau width. This value was measured from CT data in the present model. *L* was determined to be 65.60 mm. The specific translation distances of the loading point for each simulation condition were as follows. For 3° equivalent medial shift, the loading point was translated medially by 9.84 mm. For 6° equivalent medial shift, the loading point was translated medially by 19.68 mm. For 9° equivalent medial shift, the loading point was translated medially by 29.52 mm. For 3° equivalent lateral shift, the loading point was translated laterally by 9.84 mm. For 6° equivalent lateral shift, the loading point was translated laterally by 19.68 mm.

The mechanical-axis shift modeling method adopted in this study was the loading point translation method. This method maintained the three-dimensional geometry of the knee joint, ligament tension, and contact configuration completely unchanged. Only the load application point at the femoral end was translated synchronously in the medial-lateral direction. This translation simulated the deviation of the lower limb mechanical axis relative to the center of the tibial plateau. This method reflected a load-distribution type of mechanical-axis shift. It did not represent an anatomical deformity caused by bending deformation of the femur or tibia. The assumptions underlying this method were as follows. In mild to moderate varus or valgus deformities, the primary mechanism affecting joint contact mechanics was the medial or lateral shift of the load axis relative to the tibial plateau center. Geometric changes such as joint surface inclination or alterations in ligament tension were not the dominant factors. Therefore, only the loading point at the femoral end was translated in the medial-lateral direction. This approach allowed simulation of the changes in load distribution between the medial and lateral compartments under different mechanical-axis shift without introducing geometric confounding factors.

Under this framework, all mechanical-axis shift conditions (neutral 0°, medial shift 3°, 6°, 9°, and lateral shift 3°, 6°) were based on the same neutral three-dimensional geometric model of the knee joint. The initial ligament tension, contact configuration, and mesh discretization were kept identical across all conditions. Only the femoral loading point position was varied. This setup ensured that the mechanical changes were entirely attributed to the shift in the load axis. It excluded the interference of individual geometric differences on the results. Based on the above model, the following mechanical metrics were compared among different mechanical-axis shift conditions. These metrics included contact pressure of the knee joint, meniscal displacement, maximum principal stress, and tear gap.

### 2.7 Establishment of the cadaveric biomechanical experiment

A cadaveric knee specimen exhibiting normal mechanical alignment was first prepared, and biomechanical testing was subsequently performed according to the designed loading protocol. The experimental protocol was approved by the Medical Ethics Committee of the Second Hospital of Tangshan (IRB:(2026) Medical Ethics Bureau Pre-15).

A fresh frozen cadaveric knee joint specimen was selected for this study. The specimen included the femur, tibia, and fibula. It was obtained from a young male donor with a height of 173 cm, body weight of 72 kg, and BMI of 24.1. The anthropometric parameters of this donor closely matched those of the healthy volunteer used for finite element modeling. Only one cadaveric knee specimen was used in this experiment. This was due to the extremely limited availability of cadaveric specimens. The specimen was confirmed to be representative through clinical examination and imaging screening by an experienced clinician. It was verified to have no trauma, deformity, or other pathological alterations. The following morphological parameters were measured based on the specimen data. The anteroposterior (AP) diameter of the femoral condyle was 63.65 mm. The mediolateral (ML) diameter of the femoral condyle was 72.92 mm. The AP diameter of the tibial plateau was 56.76 mm. The ML diameter of the tibial plateau was 75.71 mm. The hip-knee-ankle (HKA) angle was 0.62°. Before the experiment, a senior orthopedic surgeon performed arthroscopic examination and physical examination of the specimen. The physical examinations included the drawer test, Lachman test, and varus-valgus stress test. These examinations confirmed that the medial meniscus, lateral meniscus, and major ligaments were intact. The major ligaments included the anterior cruciate ligament, posterior cruciate ligament, medial collateral ligament, and lateral collateral ligament. No tears or degenerative changes were observed.

The specimen preparation was performed by a senior orthopedic surgeon. The skin around the knee joint was first removed. The muscles of the thigh and calf were carefully dissected to expose the bone surfaces for subsequent fixation. The middle and upper segments of the femur and the middle segments of the tibia and fibula were preserved for fixture clamping. The medial collateral ligament, lateral collateral ligament, anterior cruciate ligament, posterior cruciate ligament, and patellar ligament were carefully preserved. Finally, the posterior joint capsule was incised to allow insertion of the Fuji pressure-sensitive film for contact data acquisition.

The prepared specimen was mounted on a universal mechanical testing machine using customized fixtures. The machine height was adjusted to accommodate the specimen. Prior to use, the pressure-sensitive film was trimmed to a size slightly larger than the medial and lateral compartment areas of the tibial plateau. For ease of positioning and to prevent joint fluid infiltration, the film was sealed in a specially made thin polyethylene bag. The bagged film was inserted into the joint space through the posterior capsular incision. During insertion, the knee was flexed and extended with gentle varus and valgus assistance. This allowed the film to smoothly adhere to the cartilage surface of the tibial plateau. Full coverage of the target contact area was ensured. After the film was properly positioned, the polyethylene bag edges were loosely fixed to the adjacent capsular tissue using non-absorbable sutures. This fixation served only to prevent rotation or slippage of the film during loading. It did not restrict the film from deforming under load. The central measurement area of the film, which covered the tibial plateau articular surface, remained free. It underwent compliant deformation with the joint surface during loading.

This study used Fuji Prescale pressure-sensitive film (ultra-low pressure type, *LLLW* grade, measurement range 0.2–0.6 MPa) for contact area measurement. This grade was the most sensitive level provided by Fuji. It was suitable for low-stress contact measurement at soft tissue interfaces such as cartilage and meniscus. It could effectively capture the contact area boundaries of the tibiofemoral articular surface. Before use, the film was stored in a standard environment (20–25 °C) and humidity (40%–60%) away from light. This ensured the stability of the color development reaction.

Two preliminary experiments were conducted before the formal experiment. The first preliminary experiment was performed to determine the optimal film trimming size and insertion pathway. The second preliminary experiment was performed to verify the feasibility of the loading protocol. The loading parameters included a preload of 20 N, a loading rate of 20 N/s, a peak load of 1150 N, and a holding time of 1 min. This preliminary experiment also confirmed the stability of color development after the film was kept static for 20 min following removal. The preliminary results demonstrated good repeatability of the loading protocol. No film displacement or folding was observed. The formal experiment was then carried out. The formal experiment was repeated three times. A new film was used for each experiment. The film was replaced and the specimen position was readjusted between experiments. The experimental setup is shown in Figure 6. After loading, the film was removed and kept static for 20 min. After the color development reaction was completely stabilized, each pressure film was scanned and measured three times technically using Image software. The average value of the three technical replicates was calculated. The results of the three formal experiments were expressed as mean ± standard deviation. This standard deviation reflected the measurement variability among the three repeated experiments on the same specimen. It represented experimental operational variability rather than biological variability among different individuals. These data were used for comparison with the finite element model predictions.

**FIGURE 6:**
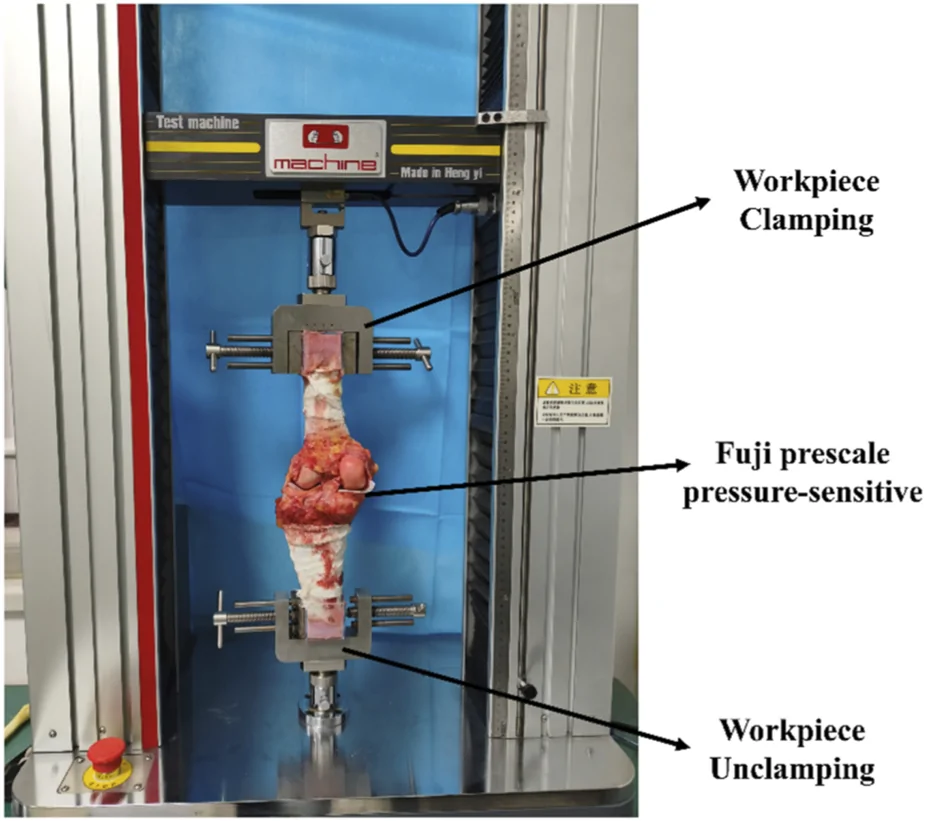
Knee cadaver experiment.

## 3 Results

### 3.1 Verification of the validity of the finite element model

Figure 7 presents the compartment contact area maps of the knee joint under a static axial load of 1150 N, obtained from both the biomechanical experiment and the finite element model. Quantitative analysis revealed that the medial compartment contact area predicted by the finite element model was 535.51 mm^2^ (Figure 7a), accounting for 41.77% of the total medial compartment area. In comparison, the medial compartment contact area measured from the biomechanical cadaveric experiment was 567.98 ± 33.43 mm^2^ (Figure 7b), representing 41.81% of the total medial compartment area. The discrepancy in medial compartment contact area between the biomechanical experiment and the finite element model was 5.72%.

**FIGURE 7:**
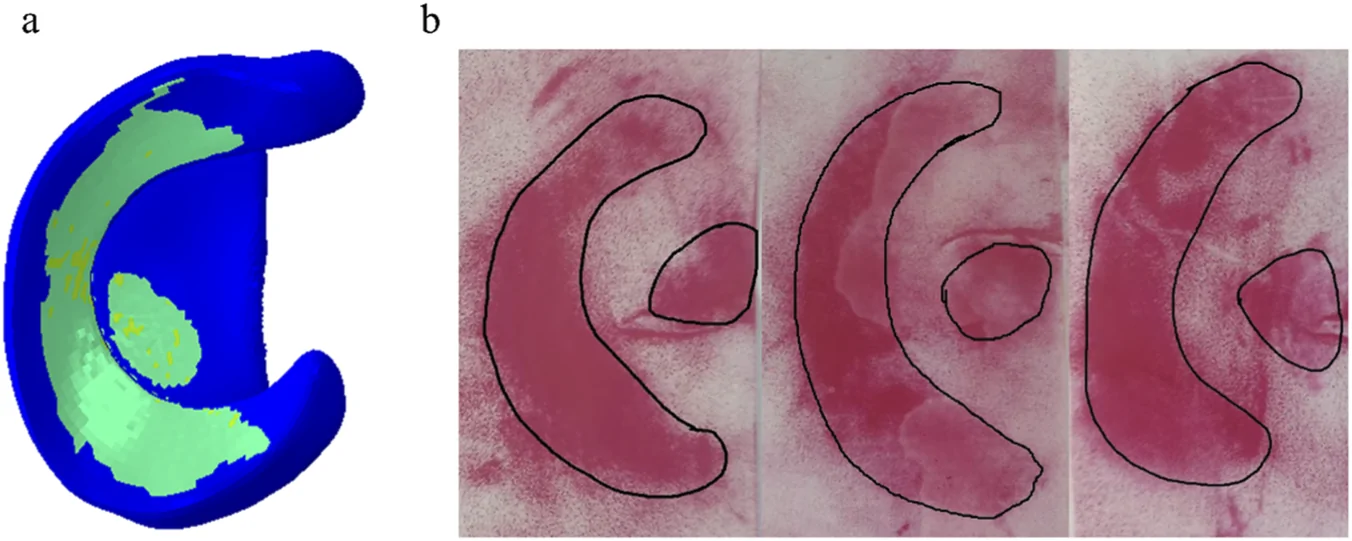
Contact distribution maps of the tibiofemoral compartments. **(a)** Contact distribution of the medial compartment predicted by the finite element model; **(b)** Contact distribution of the medial compartment obtained from the biomechanical experiment (In the three experiments, the black line represents the outline of the contact area).

### 3.2 Peak contact pressure analysis

#### 3.2.1 Neutral alignment analysis

Throughout the gait cycle (GC), the peak contact pressures in the knee components exhibited two distinct peaks, occurring at GC14 and GC44, respectively. In the intact knee model, the meniscus bore the highest contact pressure, followed by the tibial cartilage, while the femoral cartilage carried the least. Between GC14 and GC44, the lateral meniscus sustained higher contact pressures than its medial counterpart, as illustrated in Figure 8.

**FIGURE 8:**
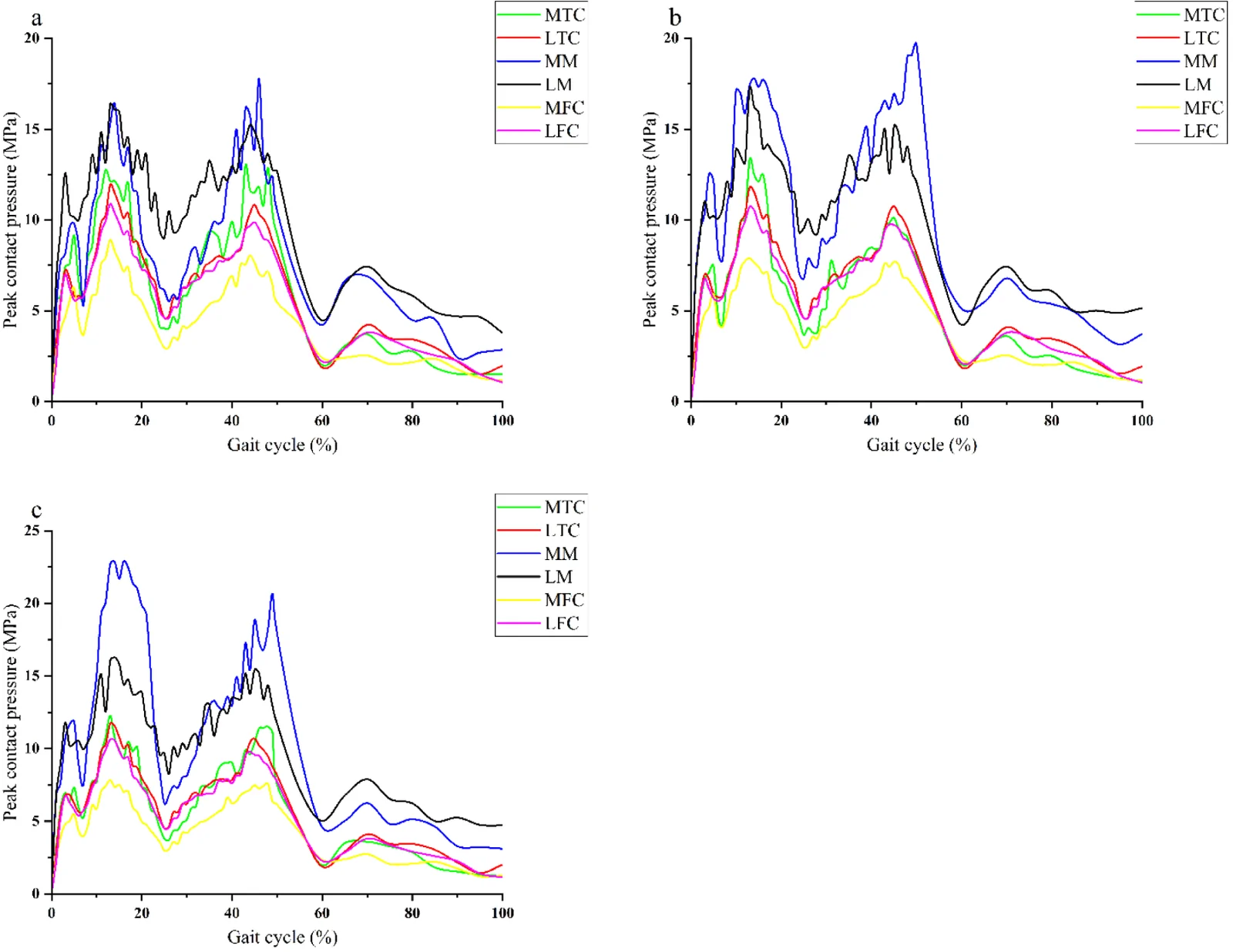
Peak contact pressure of the intact and torn menisci. Variation trends in peak contact pressure of each knee structure during the gait cycle for the intact and torn meniscus models in the neutral position. **(a)** Intact meniscus; **(b)** Stable MMPRRT; **(c)** Unstable MMPRRT. MTC, Medial tibial cartilage; LTC, Lateral tibial cartilage; MM, Medial meniscus; LM, Lateral meniscus; MFC, Medial femoral cartilage; LFC, Lateral femoral cartilage.

This study analyzed the peak contact pressure of each knee component at GC14, one of the peak points. Following a stable radial root tear in the medial meniscus, the peak contact pressure of the medial meniscus at GC14 increased from 16.42 MPa (intact) to 17.82 MPa. Under the same condition, the medial tibial cartilage pressure rose from 12.14 MPa to 12.51 MPa, while the medial femoral cartilage pressure decreased from 8.27 MPa to 7.65 MPa. In the case of an unstable radial root tear, the medial meniscal pressure at GC14 increased more substantially to 22.85 MPa. Conversely, the medial tibial cartilage pressure decreased to 10.73 MPa, and the medial femoral cartilage pressure declined to 7.35 MPa. The peak contact pressures observed in the knee components at GC14 are summarized in Table 6.

**TABLE 6:** Peak contact pressure of various components of the knee joint at GC14 (unit/MPa).

Type	Gait cycle	MTC	MM	MFC	LTC	LM	LFC
Intact	GC14	12.14	16.42	8.27	11.47	16.08	10.37
Stable	GC14	12.51	17.82	7.65	11.53	16.24	10.54
Unstable	GC14	10.73	22.85	7.35	11.55	16.23	10.55

Across the entire gait cycle, the peak contact pressure in the unstable MMPRRT model was notably higher than that in both the intact and stable MMPRRT models. The presence of a larger tear led to a redistribution of load, with a greater proportion of contact pressure shifted onto the meniscus, resulting in reduced pressures on the tibial and femoral cartilages. The medial meniscal root tear primarily influenced the peak contact pressures of the medial compartment components, with negligible effects on the lateral side.

#### 3.2.2 Analysis under medial mechanical-axis shift

As shown in Figure 9a, the peak contact pressures in the medial meniscus, medial tibial cartilage, and medial femoral cartilage exhibited maxima around GC14 and GC44. The peak contact pressures of these three structures increased with the progression of equivalent varus angle from 0° to 9°. Specifically, the peak contact pressure on the medial meniscus increased linearly with the equivalent varus angle at GC14 (*R* ^2^ = 0.9997), GC44 (*R* ^2^ = 0.9678). In contrast, the peak contact pressure on the medial femoral cartilage at GC14 and GC44 demonstrated a quadratic relationship with the equivalent varus angle, indicating an accelerating rate of pressure increase (Figures 9d,e).

**FIGURE 9:**
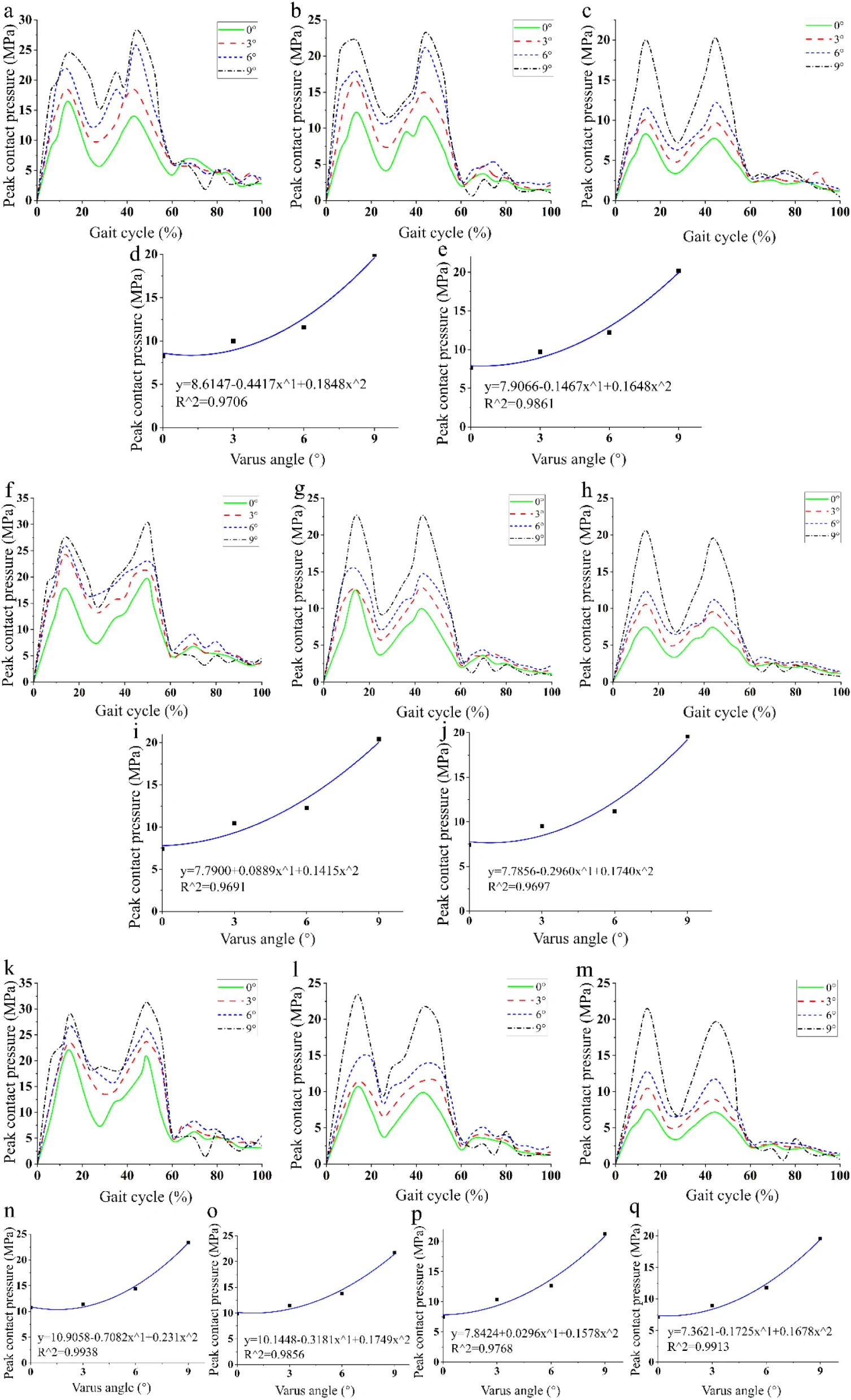
Variation trends in peak contact pressure of knee structures throughout the gait cycle under medial mechanical-axis shift. **(a)** Intact medial meniscus; **(b)** Intact medial tibial cartilage; **(c)** Intact medial femoral cartilage; **(d)** Variation trends in intact medial femoral cartilage peak contact pressure with equivalent varus angle at GC14; **(e)** Variation trends in intact medial femoral cartilage peak contact pressure with equivalent varus angle at GC44; **(f)** Stable MMPRRT medial meniscus; **(g)** Stable MMPRRT medial tibial cartilage; **(h)** Stable MMPRRT medial femoral cartilage; **(i)** Variation trends in stable MMPRRT medial femoral cartilage peak contact pressure with equivalent varus angle at GC14; **(j)** Variation trends in stable MMPRRT medial femoral cartilage peak contact pressure with equivalent varus angle at GC44; **(k)** Unstable MMPRRT medial meniscus; **(l)** Unstable MMPRRT medial tibial cartilage; **(m)** Unstable MMPRRT medial femoral cartilage; **(n)** Variation trends in unstable MMPRRT medial tibial cartilage peak contact pressure with equivalent varus angle at GC14; **(o)** Variation trends in unstable MMPRRT medial tibial cartilage peak contact pressure with equivalent varus angle at GC44; **(p)** Variation trends in unstable MMPRRT medial femoral cartilage peak contact pressure with equivalent varus angle at GC14; **(q)** Variation trends in unstable MMPRRT medial femoral cartilage peak contact pressure with equivalent varus angle at GC44.

Table 7 shows that at GC28 (i.e., the point of 1100 N static load application), the peak contact pressure of the medial knee structures increased linearly with increasing equivalent varus angle. For every 1° increase in equivalent varus angle, the peak pressure of the medial tibial cartilage, medial meniscus, and medial femoral cartilage increased by 0.79 MPa, 1.06 MPa, and 0.42 MPa, respectively.

**TABLE 7:** Peak contact pressure of the medial knee structures in the intact knee at GC28 under different equivalent varus angles.

Equivalent varus angle	Gait cycle	MTC/MPa	MM/MPa	MFC/MPa
0°	GC28	4.36	5.66	3.42
3°	GC28	7.40	9.88	4.84
6°	GC28	10.79	12.95	6.32
9°	GC28	11.49	15.20	7.21
Increase pressure/°	​	0.79	1.06	0.42

MTC, medial tibial cartilage; MM, medial meniscus; MFC, medial femoral cartilage.

As shown in Figures 9f–h and Figures 9k–m, the peak contact pressure points of the medial knee structures in both the stable and unstable MMPRRT models coincided with those in the intact meniscus model.

Stable MMPRRT: With increasing equivalent varus angle (0°–9°), the peak contact pressures of all three medial knee structures increased. The peak contact pressures of the medial meniscus and medial tibial cartilage exhibited irregular increases at GC14 and GC44. In contrast, the peak contact pressure of the medial femoral cartilage followed a quadratic relationship with equivalent varus angle (Figures 9i,j).

Unstable MMPRRT: Similarly, with increasing equivalent varus angle (0°–9°), the peak contact pressures of all three medial knee structures increased. The peak contact pressure of the medial meniscus increased linearly with equivalent varus angle at both GC14 and GC44 (*R* ^2^ = 0.9869 and *R* ^2^ = 0.9740, respectively). The peak contact pressures of the medial tibial cartilage and medial femoral cartilage followed quadratic relationships with equivalent varus angle (Figures 9n–q).

### 3.3 Meniscal displacement analysis

Two meniscal displacement metrics were used to characterize the kinematic behavior of the meniscus in this study. Meniscal extrusion displacement was defined as the outward displacement of the outer edge of the meniscus relative to the tibial plateau edge in the coronal plane. This metric reflected the degree of radial extrusion of the meniscus in the medial-lateral direction. Overall meniscal displacement was defined as the resultant displacement vector of the meniscus in three-dimensional space. This metric characterized the total displacement magnitude of the meniscus in space without distinguishing direction. Both metrics were calculated based on the tibial plateau coordinate system. The origin was set at the geometric center of the tibial plateau. The displacement data were extracted at the midpoint of the outer edge of the medial meniscus. This point was selected to represent the representative displacement behavior of the tissue surrounding the tear.

#### 3.3.1 Neutral alignment meniscal extrusion analysis

Figure 10 illustrates the meniscal extrusion displacement throughout the gait cycle for both the intact and torn menisci. The maximum extrusion magnitudes occurred at GC14, GC44, and GC88. Medial meniscus posterior root tears resulted in increased extrusion displacement, with the unstable tear model exhibiting the greatest extrusion (2.69 mm at GC44).

**FIGURE 10:**
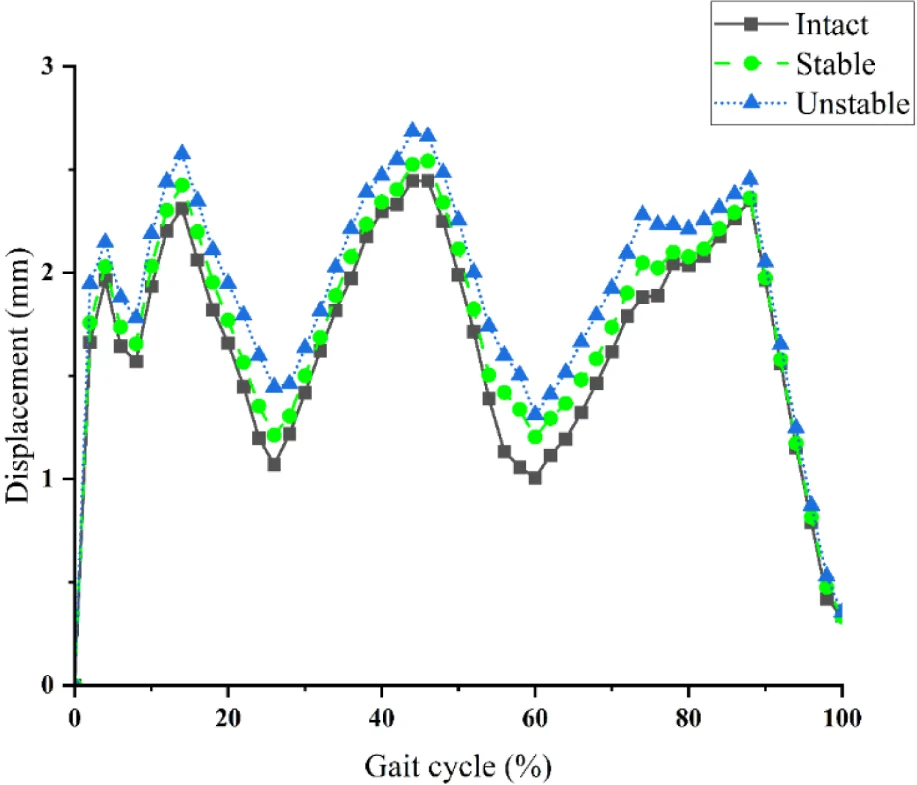
Variation trends of meniscal extrusion displacement throughout the gait cycle in the intact and torn menisci under neutral alignment.

#### 3.3.2 Analysis of overall meniscal displacement under medial mechanical-axis shift

Figures 11a–c illustrates the overall displacement trends of the intact and torn menisci throughout the gait cycle under medial mechanical-axis shift. Under medial mechanical-axis shift, the displacement trends of the intact and torn menisci were generally consistent.

**FIGURE 11:**
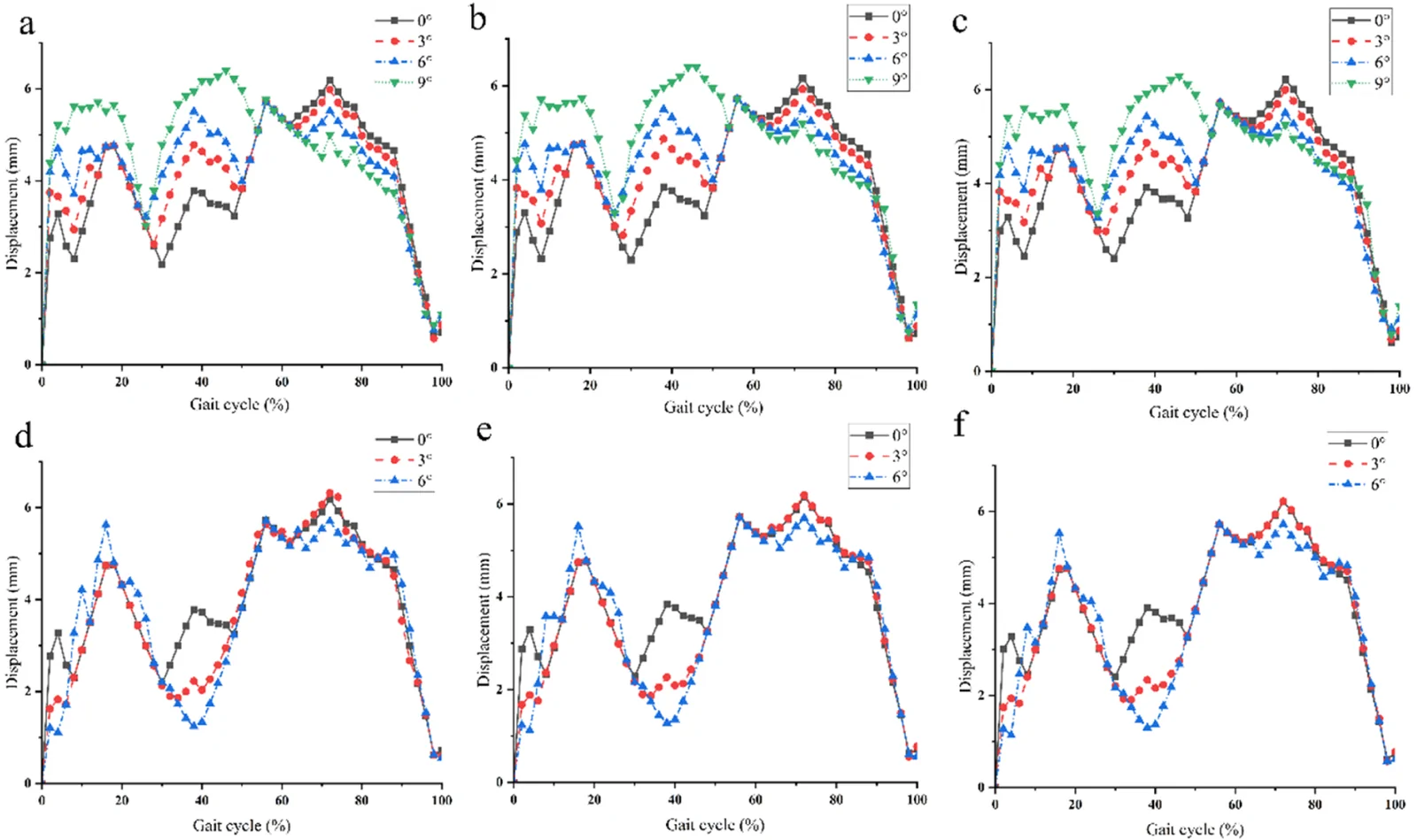
Variation trends of overall meniscal displacement throughout the gait cycle in intact and torn menisci. **(a)** Intact meniscus under medial mechanical-axis shift; **(b)** Stable MMPRRT under medial mechanical-axis shift; **(c)** Unstable MMPRRT under medial mechanical-axis shift; **(d)** Intact meniscus under lateral mechanical-axis shift; **(e)** Stable MMPRRT under lateral mechanical-axis shift; **(f)** Unstable MMPRRT under lateral mechanical-axis shift.

From GC0 to GC56, meniscal displacement increased with increasing equivalent varus angle. The greater the equivalent varus angle, the longer the duration of high meniscal displacement within the gait cycle, which was most pronounced from GC26 to GC54 (0°: GC30-GC50; 3°: GC28-GC50; 6°: GC26-GC52; 9°: GC26-GC54).

From GC62 to GC88, meniscal displacement decreased with increasing equivalent varus angle. A smaller equivalent varus angle was associated with a longer duration of high meniscal displacement during this period (0°: GC62-GC88; 3°: GC64-GC88; 6°: GC66-GC88; 9°: GC70-GC88).

From GC88 to GC100, meniscal displacement remained largely consistent across different equivalent varus angles.

#### 3.3.3 Analysis of overall meniscal displacement under lateral mechanical-axis shift

Figures 11d–f illustrates the overall displacement trends of the intact and torn menisci throughout the gait cycle under lateral mechanical-axis shift. Under lateral mechanical-axis shift, the displacement trends of the intact and torn menisci were generally consistent.

From GC30 to GC50, meniscal displacement at neutral 0° alignment was higher than that under lateral mechanical-axis shift.

From GC62 to GC88, meniscal displacement decreased with increasing equivalent valgus angle. A smaller equivalent valgus angle was associated with a longer duration of high meniscal displacement during this period (0°: GC62-GC88; 3°: GC64-GC88; 6°: GC66-GC88).

From GC88 to GC100, meniscal displacement remained largely consistent across different equivalent valgus angles.

### 3.4 Meniscal tear gap analysis

#### 3.4.1 Neutral alignment analysis

The tear gap sizes for stable and unstable MMPRRTs throughout the gait cycle are shown in Figure 12. Under gait loading, the unstable tear exhibited a notably larger gap than the stable tear. Peak gap values occurred at GC14 and GC44, with the maximum observed at GC14. At this point, the gap measured 2.40 mm for the stable tear and 3.57 mm for the unstable tear, representing a 48.75% increase.

**FIGURE 12:**
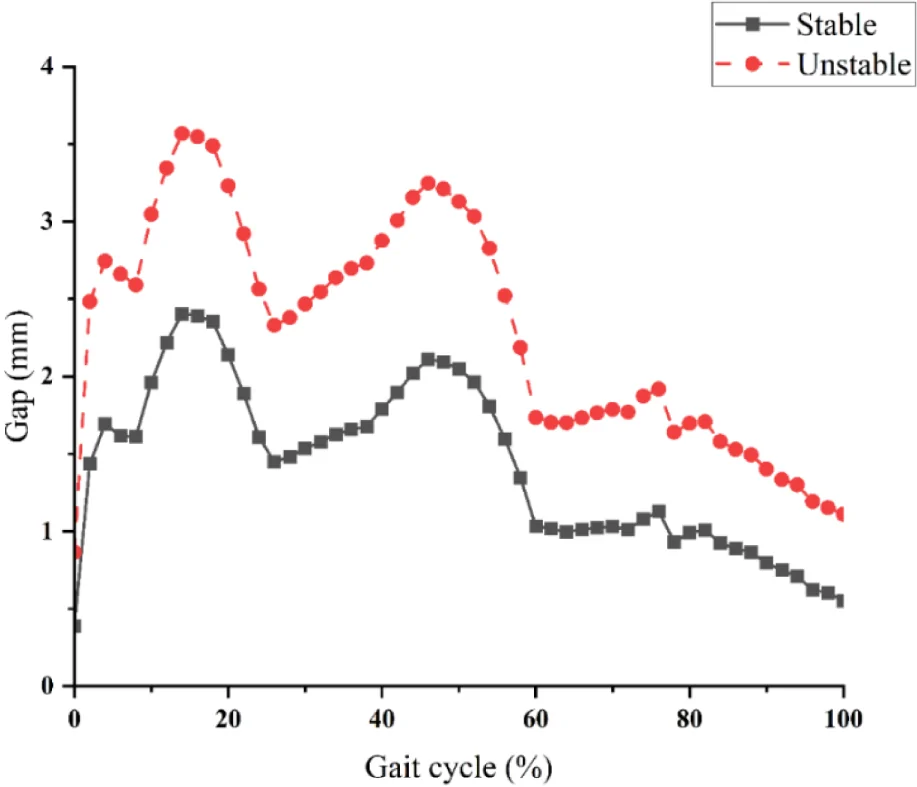
Variation trends of the meniscal tear gap throughout the gait cycle under neutral alignment.

#### 3.4.2 Analysis under medial and lateral mechanical-axis shifts

As shown in Figures 13a,b, under medial mechanical-axis shift conditions, the first peak tear gap occurred between GC14 and GC16 in all models. The second peak appeared at GC46 in the 3° and 6° medial shift models, and at GC52 in the 9° medial shift models. When the varus angle ranged from 0° to 6°, the tear gap exhibited an increasing trend with increasing equivalent varus angle, although the rate of increase diminished. In the GC14 phase, when the equivalent varus angle increased from 0° to 3°, the stable MMPRRT gap increased by 15% (0.36 mm) and the unstable MMPRRT gap increased by 11.5% (0.41 mm); when the equivalent varus angle increased from 3° to 6°, the stable MMPRRT gap increased by 6.2% (0.16 mm) and the unstable MMPRRT gap increased by 4.5% (0.20 mm). When the equivalent varus angle ranged from 6° to 9°, the MMPRRT gap exhibited a decreasing trend during certain phases of the gait cycle, but the tear gap remained at a persistently high level.

**FIGURE 13:**
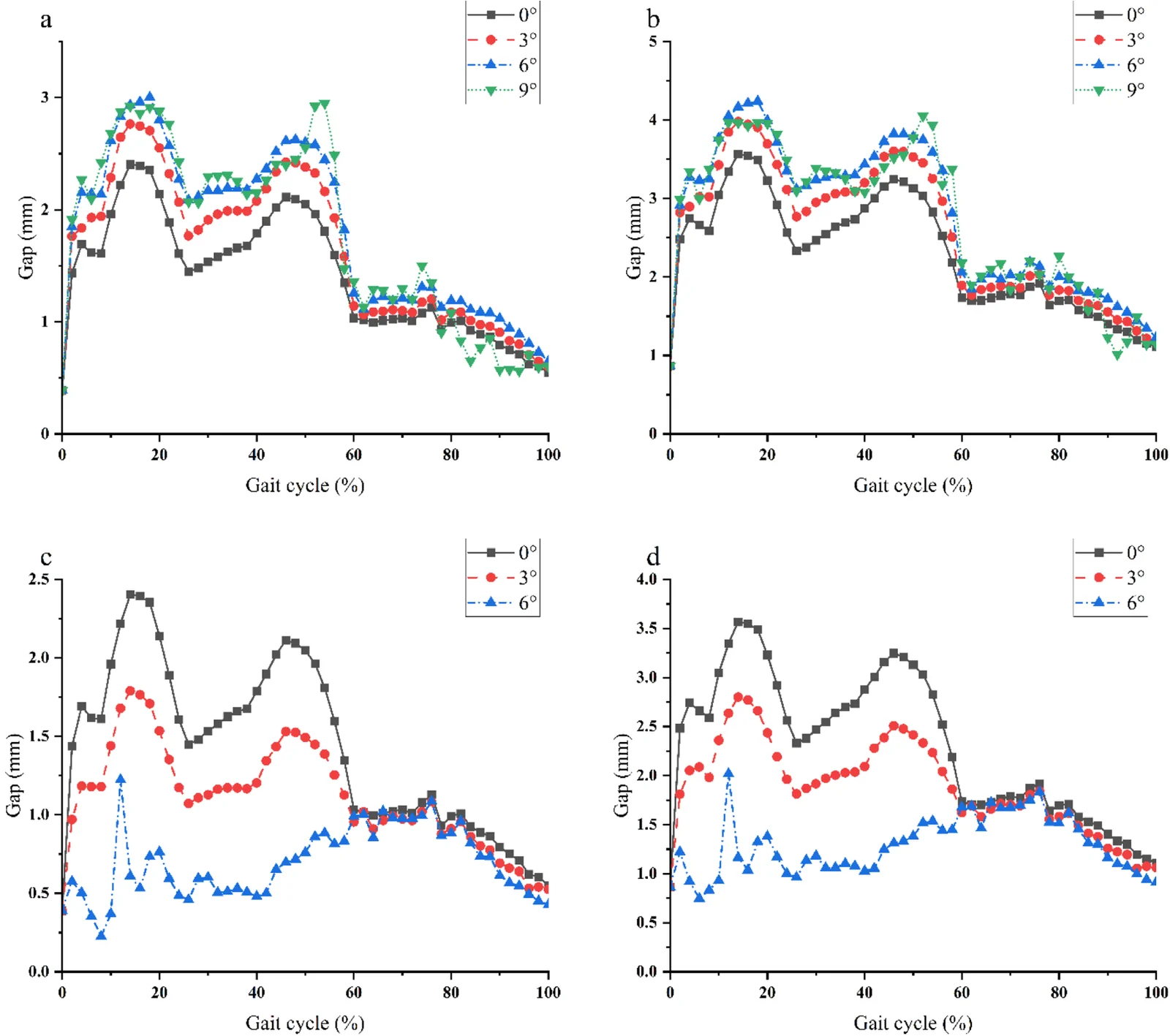
Variation trends of the meniscal tear gap throughout the gait cycle under medial and lateral mechanical-axis shifts. **(a)** Stable MMPRRT under medial mechanical-axis shift; **(b)** Unstable MMPRRT_ under medial mechanical-axis shift; **(c)** Stable MMPRRT under lateral mechanical-axis shift; **(d)** Unstable MMPRRT under lateral mechanical-axis shift.

As shown in Figures 13c,d, under lateral mechanical-axis shift conditions, the two peak tear gaps in the 3° lateral shift model occurred at GC14 and GC46, respectively, while those in the 6° lateral shift model occurred at GC12 and GC76. When the equivalent valgus angle ranged from 0° to 6°, the MMPRRT gap exhibited a decreasing trend with increasing equivalent valgus angle, indicating a model-predicted reduction in tear gap under lateral mechanical-axis shift.

### 3.5 Maximum principal stress analysis

#### 3.5.1 Neutral alignment analysis

As shown in Figure 14a, across the entire gait cycle, the maximum principal stress in the meniscus was markedly higher in the stable MMPRRT model than in the unstable MMPRRT model, with the peak value of 61.45 MPa occurring at GC14. The radial tear disrupted the transmission of hoop stress, resulting in stress concentration at the tear apex. Figures 14b,c illustrates the maximum principal stress distribution in the torn meniscus. The maximum principal stress was predominantly concentrated at the tear apex and in the anterior portion of the meniscus. In the stable radial tear model, maximum principal stress was present on both sides of the tear apex, but with opposite directions. In contrast, in the unstable MMPRRT model, maximum principal stress was observed only on one side of the apex and exhibited a sparse distribution, which may account for the lower stress levels observed in the unstable MMPRRT model.

**FIGURE 14:**
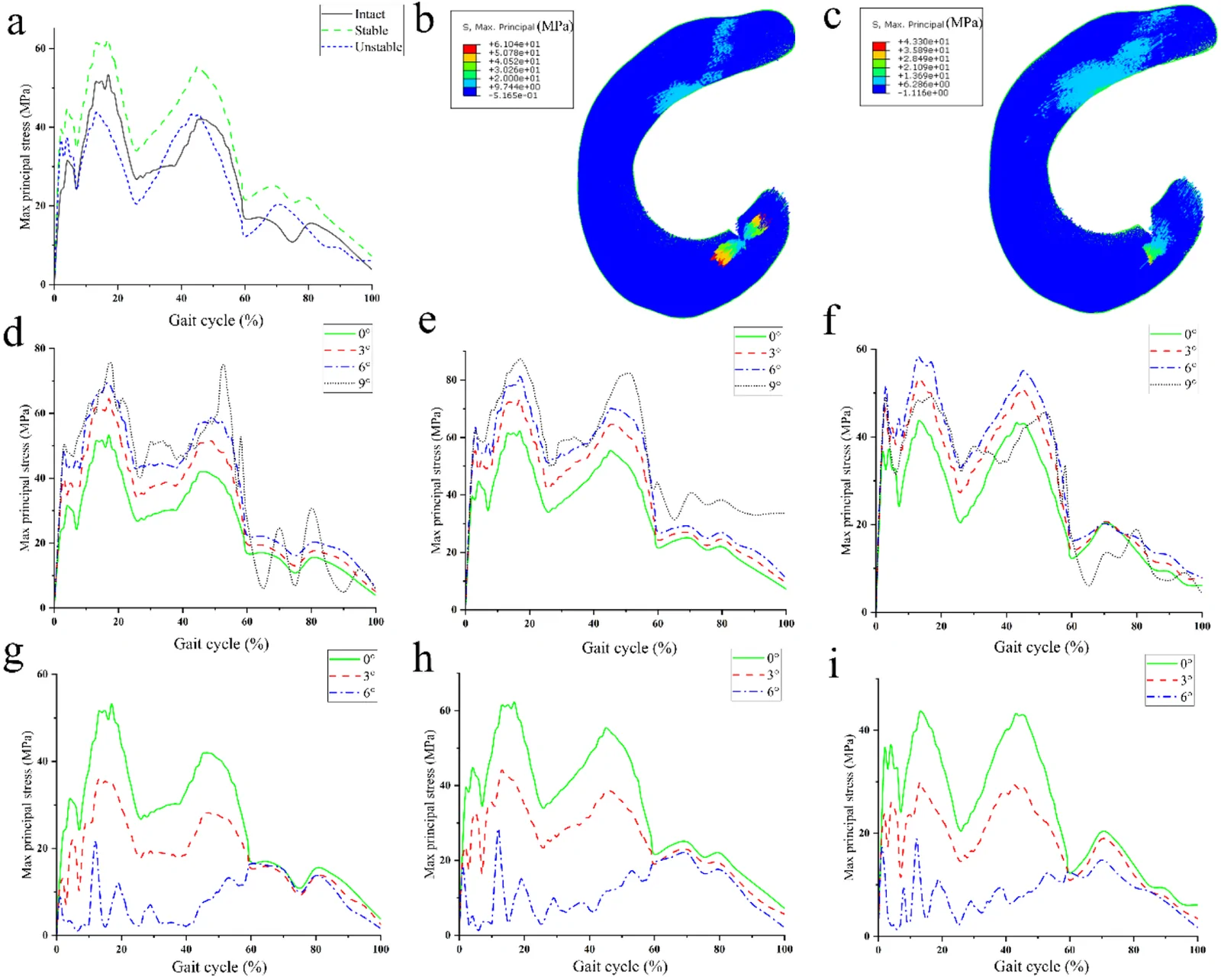
Maximum principal stress distribution in the torn meniscus and its variation trends throughout the gait cycle. **(a)** Intact and torn menisci under neutral alignment; **(b)** Maximum principal stress tensor distribution in the stable MMPRRT; **(c)** Maximum principal stress tensor distribution in the unstable MMPRRT (red arrows indicate the direction of maximum principal stress, with stress concentration at the tear apex); **(d)** Intact meniscus under medial mechanical-axis shift; **(e)** Stable MMPRRT under medial mechanical-axis shift; **(f)** Unstable MMPRRT under medial mechanical-axis shift; **(g)** Intact meniscus under lateral mechanical-axis shift; **(h)** Stable MMPRRT under lateral mechanical-axis shift; **(i)** Unstable MMPRRT under lateral mechanical-axis shift.

#### 3.5.2 Analysis under medial mechanical-axis shift

As shown in Figures 14d–f, the maximum principal stress in both the intact medial meniscus and the stable MMPRRT model increased with increasing equivalent varus angle. For the unstable MMPRRT model, the maximum principal stress increased with equivalent varus angle in the early stage of medial shift (0°–6°). However, at 9° medial shift, the peak maximum principal stress decreased markedly, falling below the value observed at 3°. Overall, under medial mechanical-axis shift, the maximum principal stress in the stable MMPRRT model remained higher than that in both the intact meniscus and the unstable MMPRRT model.

#### 3.5.3 Analysis under lateral mechanical-axis shift

Under lateral mechanical-axis shift, the maximum principal stress in the intact medial meniscus and in both the stable and unstable MMPRRT models exhibited consistent trends throughout the gait cycle, all decreasing with increasing equivalent valgus angle. Compared with the 0°–3° lateral shift range, a pronounced reduction in the maximum principal stress was observed in the 3°–6° lateral shift range. The relevant results are presented in Figures 14g–i.

## 4 Discussion

This study established a dynamic knee joint model with mechanical-axis shift. The results demonstrated that mechanical-axis shift was an important factor causing biomechanical changes in the knee joint. Medial mechanical-axis shift led to increased pressure at the posterior root of the medial meniscus tear. It also resulted in increased cartilage pressure under dynamic loading. This may contribute to the development of osteoarthritis. Previous studies have investigated the relationship between lower limb alignment and medial compartment biomechanics. However, the biomechanical changes of the knee joint with mechanical-axis shift under dynamic gait loading remain less well characterized.

The accuracy of the model was evaluated by comparing the predictions of the intact knee joint model with the experimental data from the cadaveric knee specimen. The difference in medial compartment contact area between the biomechanical experiments and the finite element model was 5.72%. The contact areas obtained from the two methods showed good consistency. To further evaluate the plausibility of the model predictions, the predicted peak contact pressures were compared with the results of finite element and experimental studies reported in the literature. The comparisons were made in terms of trends (Table 8). Differences existed among studies in model geometry, material properties, loading conditions, and result definitions. Therefore, this comparison should be considered as an external plausibility assessment of the present model. In this study, the peak contact pressure of the knee joint at GC28 increased linearly with increasing equivalent varus angle. In a previous cadaveric study, Mina et al. (Mina et al., 2008) reported that the mean contact pressure in the medial compartment increased linearly with increasing equivalent varus angle. Willinger et al. (Willinger et al., 2019) reported that a 3° varus alignment increased the medial compartment pressure by 37.4%. These findings support the mechanical plausibility of the predicted trends in the present model. Biomechanical studies have shown that approximately 40%–60% of the load acting on the knee joint is transmitted through the menisci. This includes 65%–70% of the load on the lateral side and 40%–50% on the medial side (Fox et al., 2015). The present model predicted a similar trend. In the neutral intact knee joint model, the duration of high peak contact pressure in the lateral meniscus was considerably longer than that in the medial meniscus throughout the gait cycle. In addition, the peak contact pressure observed in this study was higher than that reported in vitro experimental studies. We considered that the main reason for this difference was that the pressure sensors used in the *in vitro* experiments were placed between the meniscus and the tibial cartilage. These sensors could not capture the peak force between the meniscus and the femoral cartilage.

**TABLE 8:** Validation of peak contact pressure in the finite element model.

Study	State	MTC/MPa	MM/MPa	MFC/MPa
Wang et al. (2022)	GC14	11.94	​	​
	GC45	12.45	​	​
Mao et al. (2025)	GC14	6.6	14.0	11.0
	GC44	10.0	9.8	10.4
Xu et al. (2022)	Flexion 0°	Peak contact pressure in the medial compartment: 7.9 MPa		
Thambyah (2007)	1144N	Peak contact pressure in the medial compartment: 6–11 MPa		
Our study	GC14	12.14	16.42	8.27
	GC28 (1100N)	4.36	5.66	3.42
	GC44	11.64	15.75	8.04

This table summarizes the peak contact pressures of the intact knee joint under neutral alignment with static or dynamic loading conditions reported in different studies. Differences existed among studies in model construction methods, loading conditions, material parameters, and result definitions. Therefore, this table was used only to assess whether the predictions of the present model fell within the reasonable range reported in the literature.

MTC, medial tibial cartilage; MM, medial meniscus; MFC, medial femoral cartilage.

In addition, the simulation results showed that the torn meniscus exhibited greater extrusion displacement during the gait cycle. The longer the tear, the greater the displacement. This finding was consistent with the results of static simulations of radial meniscal tears. In a static study, Zhang et al. (Zhang et al., 2019) found that radial tears of the medial meniscus led to greater meniscal extrusion. Clinical studies have shown that medial meniscus root tears were significantly associated with increased meniscal extrusion (Costa et al., 2004; Muzaffar et al., 2015). Furumatsu et al. (Furumatsu et al., 2017) demonstrated that even in the early stage, the mean absolute medial extrusion was 3.0 ± 1.2 mm. This occurred within 1 month after the onset of symptomatic MMPRT. Medial extrusion progressed gradually within 1 year after symptom onset. The meniscal extrusion displacement predicted by the present model was consistent with these clinical measurements. However, clinical MRI measurements were typically performed under static non-weight-bearing or partial-weight-bearing conditions. The extrusion displacement in this study was obtained from dynamic gait simulations. The loading conditions between the two were fundamentally different. Therefore, this comparison was used only to evaluate the clinical plausibility of the model predictions. Radial tears disrupted the circumferential collagen fibers. This ultimately led to pathological meniscal extrusion (Papalia et al., 2013).

The overall meniscal displacement increased with increasing equivalent varus angle. A larger equivalent varus angle resulted in a longer duration of high meniscal displacement throughout the gait cycle. This indicated that the meniscus remained in a state of increased mobility for a greater proportion of the gait cycle. Crema et al. (Crema et al., 2012) and Lefevre et al. (Lefevre et al., 2016) demonstrated that varus alignment was an independent factor for medial meniscal extrusion. Our study confirmed this finding from a biomechanical perspective. We considered that under medial mechanical-axis shift, the meniscus was subjected to prolonged high displacement. This may have exposed the meniscal tissue to repeated tensile and shear stresses. Such mechanical conditions may have reduced the elastic modulus and tensile strength of the tissue. The abnormal mechanical environment may have stimulated elevated expression of inflammatory cytokines in the meniscal tissue. This may have led to tissue degradation and degenerative changes. Ultimately, this may have resulted in pathological extrusion (Papalia et al., 2013).

This study examined how mechanical-axis shift affects meniscal stress distribution during the dynamic gait cycle. With increasing equivalent varus angle, the peak contact pressure of the medial tibial cartilage, medial meniscus, and medial femoral cartilage all showed an increasing trend. However, the response patterns of each structure differed across different gait cycle time points. The peak contact pressure of the medial meniscus at GC14 increased linearly with equivalent varus angle. The peak contact pressure of the medial femoral cartilage at GC14 increased in a quadratic curve pattern. At GC28, it increased linearly. These findings suggested that the load-bearing mechanisms of each tissue may differ across phases of the gait cycle. The non-physiological loading characteristics of cartilage tissue under abnormal alignment may be associated with accelerated cartilage degeneration in patients with varus deformity (Palmer et al., 2020). However, the specific mechanisms require further investigation.

Medial mechanical-axis shift not only affected contact stress but also regulated the morphology of the meniscal tear gap and the distribution of maximum principal stress. As shown in Figure 13, within the medial shift range of 0°–6°, the radial tear gap increased with increasing equivalent varus angle. However, the rate of increase gradually slowed. Within the range of 6°–9°, the tear gap showed a decreasing trend. The results in Figures 14d–f showed that the maximum principal stress in the intact meniscus and the stable MMPRRT increased with increasing equivalent varus angle. The rate of increase gradually slowed. For the unstable MMPRRT, the maximum principal stress increased in the early stage of varus (0°–6°) but decreased at 6°–9°. These findings indicated that the mechanical response of the knee joint under medial mechanical-axis shift exhibited complex nonlinear characteristics. At smaller equivalent varus angles (0°–6°), the tension on the circumferential fibers of the meniscus continued to increase. The tear zone was stretched. Collagen fibers may have gradually ruptured and ductility may have decreased. The resistance to gap expansion increased, and the growth rate slowed. At this stage, the remaining fibrous structure could still bear part of the load. When the equivalent varus angle further increased (6°–9°), the reduction in tear gap may have been associated with severe meniscal extrusion. At this point, the tension transmission function of the circumferential fibers may have been considerably weakened. Effective hoop stress could not be generated. The load could not be effectively dispersed through the meniscus. These trends suggested that the effect of equivalent varus angle on meniscal mechanical behavior exhibited stage-dependent differences in the present model predictions. However, the specific inflection points and underlying mechanisms require further validation through additional data points and experimental studies.

MMPRRT is a common injury of the knee joint (Bhatia et al., 2014; Jeon et al., 2022; Steineman et al., 2022). Unstable tears are of particular clinical concern. During the gait cycle, the peak contact pressure in the unstable MMPRRT model was considerably higher than that in the intact and stable models. The tear gap in the unstable model was also considerably larger than that in the stable tear. With increasing tear size, more contact pressure was transferred to the meniscus. This led to reduced contact pressure on the tibial and femoral cartilage. This phenomenon indicated that a larger radial tear caused more severe disruption of the circumferential fiber continuity at the meniscal posterior root. The local stiffness of the meniscus may have decreased and deformation may have increased. The load became more concentrated in the meniscal tissue itself. This reflected a local redistribution of load between the meniscus and cartilage. In the presence of medial mechanical-axis shift, the overall increase in total load on the medial compartment may have masked this local change. Ultimately, cartilage pressure still increased with increasing equivalent varus angle. In addition, the maximum principal stress in the stable MMPRRT was considerably higher than that in the unstable MMPRRT. As shown in Figures 14b,c, the stress distribution in the stable tear exhibited opposite directions and similar stress density on both sides of the tear. In the unstable tear, high-density stress was concentrated only on one side of the tear. These differences in stress distribution suggested that tear size may affect the mechanobiological environment of the meniscus by altering the local stress field. However, the relationship between tear size and tear progression requires further investigation.

### 4.1 This study had several limitations

First, model validation was based on static loading experiments using one cadaveric knee specimen. Only contact area was compared. Due to limited specimen availability, validation was performed only under neutral alignment (0°) with static axial loading. Validation under multiple angles, multiple loading conditions, and dynamic loading had not been performed. The pressure-sensitive film method used in the experiment also had inherent limitations. These included capsular incision and the effect of film thickness on contact area measurement. Therefore, the current validation could only partially support the predictive reliability of the model under specific conditions. The prediction accuracy under different loading conditions required further validation.

Second, this study used a single-subject knee geometric model. The effects of inter-individual differences in joint morphology, cartilage thickness, and ligament attachment positions on the mechanical response were not considered. Whether the computational results of the present model were generalizable to the general population required further validation using multi-subject models and statistical analysis.

Third, the mechanical-axis shift model in this study adopted the loading point translation method. This method reflected a load-distribution type of mechanical-axis shift. It did not represent anatomical deformity caused by bending deformation of the femur or tibia. The model did not consider joint surface inclination, changes in ligament tension, or alterations in joint kinematics. Therefore, the present findings should be interpreted as a mechanical-axis shift rather than a true anatomical deformity.

Fourth, muscle forces were not included in the present model. The ISO-14243–3 standard loading curves were used, which were based on average population gait data. Individualized muscle activation patterns and gait strategies could not be reflected. This may have affected the prediction accuracy of absolute contact pressure. However, because the loading conditions were identical across all conditions, the relative trends among different conditions remained reliable. Some studies have indicated that incorporating muscle forces is a complex task (Benos et al., 2020). Many studies have analyzed knee joint mechanics from the perspective of bones and ligaments and obtained satisfactory results. Nevertheless, future studies should establish knee models that include muscle forces and incorporate subject-specific gait data. This would allow a more comprehensive reflection of the individualized dynamic joint mechanical environment.

Fifth, the conclusions of this study were based on predictions from the finite element model. Their clinical significance required further validation through prospective clinical studies.

## 5 Conclusion

This study established a dynamic finite element model of the knee joint with mechanical-axis shift. The effects of mechanical-axis shift and tear length on the stress distribution of MMPRRT were analyzed throughout the dynamic gait cycle. It should be noted that anatomical malalignment was not directly modeled. The alignment conditions represent a mechanical-axis shift achieved by translating the loading point. In the present model, mechanical-axis shift and meniscal tear were associated with changes in tibiofemoral contact stress, meniscal displacement, tear gap, and maximum principal stress. Medial mechanical-axis shift was associated with increased medial compartment loading. Lateral mechanical-axis shift was associated with reduced medial compartment loading. These biomechanical associations predicted by the model suggested that mechanical-axis shift may play a potential role in the mechanical environment of MMPRRT. However, the clinical significance of these findings requires further validation through prospective clinical studies.

## Data Availability

The raw data supporting the conclusions of this article will be made available by the authors, without undue reservation.
